# The Application of Magnetic Resonance Imaging in the Early and Accurate Diagnosis of Hip Joint Avascular Necrosis

**DOI:** 10.7759/cureus.68888

**Published:** 2024-09-07

**Authors:** Tushar Kalekar, Kushal Bothara, Purnachandra Lamghare

**Affiliations:** 1 Radiodiagnosis, Dr. D. Y. Patil Medical College, Hospital and Research Centre, Dr. D. Y. Patil Vidyapeeth (Deemed to be University), Pune, IND

**Keywords:** avascular necrosis, ficat and arlet staging, hip joint, magnetic resonance imaging, radiograph

## Abstract

Introduction

Avascular necrosis (AVN) is characterized by the death (necrosis) of cellular bone components in the subchondral bone or epiphysis due to a lack of or an interruption of the blood supply. In routine practice, AVN is most frequently encountered in the femoral head. In this study, we aim to evaluate the application of magnetic resonance imaging (MRI) in the early and accurate diagnosis of hip joint AVN.

Materials and methods

This was a retrospective, cross-sectional study conducted in the Department of Radiodiagnosis of Dr. D. Y. Patil tertiary care hospital, Pimpri, Pune, India. We studied 30 patients with complaints of pain and associated limping who underwent primary radiograph analysis of the hip joint, followed by MRI.

Results

We assessed 30 patients (45 hip joints) using plain radiography and MRI. Of the 45 hips, we could diagnose AVN in 28 hips (62.2%) using plain radiography, but we could not diagnose it in 17 hips (37.8%), whereas we were able to diagnose AVN in all hips (100%) using MRI. Forty percent of the patients (n = 12) were on steroids, 26.7% (n = 8) were chronic alcoholics, and 16.7% (n = 5) were idiopathic. The other less common causes were a history of trauma or fracture of the neck of the femur (n = 3) and sickle cell disease (n = 2). Of the 45 hips of the 30 patients studied, 15 patients had bilateral disease affecting a total of 30 hips (66.7%), and 15 patients had unilateral disease affecting a total of 15 hips (33.4%). Of the 30 hips (bilateral disease), five (13.3%) contralateral hips were clinically occult and were incidentally diagnosed with AVN.

Conclusion

The assessment of AVN based solely on plain radiography can miss vital information in stages II and III (Ficat and Arlet classification). Due to its multiplanar capability, superior spatial resolution, and better tissue characterization, MRI is very sensitive and able to detect femoral head AVN early and promptly in cases that are radiograph-negative or otherwise clinically unsuspected.

## Introduction

The prevalence of avascular necrosis (AVN) of the hip joint (femoral head), a progressive and difficult to detect clinical disease, is rising daily [1,2]. Clinically, AVN of the femoral head is a pathological condition accompanied by a reduction in the vascular supply to the femoral head’s subchondral bone. As a result, osteocytes die, articular surfaces gradually disintegrate, and degenerative hip joint disease develops later. Nontraumatic AVN of the femoral head is often related to alcohol usage [3], the use of glucocorticoids and other steroids [4], or the presence of hematologic diseases such as sickle cell disease [5]. However, the cause of nontraumatic AVN of the femoral head is unknown in roughly 30% of patients; thus, these are referred to as idiopathic [6]. AVN becomes detectable one to six months after exposure to an easily identifiable risk factor such as high-dose glucocorticoid therapy or a fracture of the femoral neck. Thereafter, AVN is relatively uncommon, even after the patient remains exposed to the risk factor [7].

Early in the disease phase, patients may not exhibit any symptoms. However, when patients start to show symptoms, the reported history is typically of hip pain that can spread to the groin and/or thigh. Resting usually relieves the pain, whereas activities such as walking or climbing stairs usually increase it. Even when there is no movement, the pain will frequently persist [8]. In physical examinations that suggest osteonecrosis of the femoral head, the hip or groin is the predominant location of discomfort; knee pain and restricted internal hip rotation may also be present on occasion [9].

Plain radiography, magnetic resonance imaging (MRI), computed tomography (CT), skeletal scintigraphy, and single-photon emission computed tomography are currently available noninvasive diagnostic procedures for identifying AVN of the hip joint. While the overall sensitivity for early-stage AVN is only approximately 41%, radiograph-based detection is not sensitive enough to identify AVN of the femoral head at its inception (stage I) [10]. This makes it necessary for MRI to play a part in the early and precise identification of hip joint AVN. Accurate structural and pathological changes of the femoral head were shown by MRI, which is crucial for early therapy. Additionally, MRI showed bone marrow edema and an abnormal subchondral geographical area that were not seen on an X-ray but imply chronic ischemic damage.

We aimed to study the role of plain radiographs and MRI findings in the diagnosis of AVN, as well as the role of MRI in determining clinically occult contralateral AVN.

## Materials and methods

This was a retrospective, cross-sectional study conducted at the Department of Radiodiagnosis of Dr. D. Y. Patil tertiary care hospital, Pimpri, Pune, India, over a period of six months from September 2023 to February 2024. The Institutional Ethics Sub-Committee of Dr. D. Y. Patil Medical College, Hospital & Research Centre, Dr. D. Y. Patil Vidyapeeth, issued approval I.E.S.C./W/132/2024. Two radiologists with a minimum of three years of experience in conventional and cross-sectional imaging were involved in the study interpretation. We included in our study all magnetic resonance (MR)-positive cases of hip AVN that also underwent radiograph assessment. We included patients clinically suspected of AVN, patients older than 18 years, and those who underwent hip radiography, followed by MRI, and were diagnosed with femoral head AVN. We also included patients with clinically occult but MRI-diagnosed contralateral femoral head AVN. We excluded pregnant females and patients who had previously been diagnosed with femoral head AVN and had undergone medical or surgical treatment or had a history of claustrophobia, metallic implants, or cardiac pacemakers.

We performed anteroposterior hip radiography as the first step in the diagnostic evaluation of the hips clinically suspected for AVN using a Multix Select digital radiography system (Siemens Medical Solutions USA, Inc., Malvern, PA). Protocol and exposure factors were as follows: kilovoltage peak (kVp) was 70-75, milliampere-seconds (mAs) was 50-60, and film focus distance was 110 cm.

We assessed anteroposterior hip radiographs according to the Ficat and Arlet classification (Table 1).

**Table 1:** Plain radiograph images assessed according to the Ficat and Arlet classification.

Stages	Description
Stage I	Normal radiographs/minor osteopenia
Stage II	Subarticular sclerotic or cystic lesions
Stage III	Flattening or collapse of the femoral head with or without subchondral fracture (crescent sign); normal joint space
Stage IV	End-stage femoral head collapse together with secondary degenerative changes (secondary osteoarthritis)

We performed an MRI of the hips using a 3T MAGNETOM Vida MRI platform (Siemens Medical Solutions USA, Inc., Malvern, PA). The protocol for MRI was as follows: the coil used was a torso phased array coil; the patient’s position was supine, with feet first. We performed nonenhanced MR sequences on all patients using the parameters shown in Table 2.

**Table 2:** Parameters for nonenhanced MR sequences performed on all patients. T1W, T1-weighted; T2W, T2-weighted; STIR, short tau inversion recovery; MR, magnetic resonance

	Repetition time (TR) (ms)	Echo time (TE) (ms)	Field of view (FOV) (cm)	Slice thickness (mm)
T1W axial	400-600	10-20	10-16	4-6
T2W axial	3,000-4,000	70-85	10-15	4-6
T1W coronal	400-600	10-20	12-18	4-6
T2W coronal	3,000-3,500	70-75	12-18	4-6
STIR coronal	4,000-4,500	40-50	12-18	4-6

We evaluated the MRI images using both the Ficat and Arlet classification and the Association Research Circulation Osseous (ARCO) classification. We used both systems for staging AVN, wherein stage I presented with marrow edema and stage II presented with a defective or abnormal subchondral geographical area. Stage III and stage IV presented with findings similar to stage III and IV radiograph findings in Table 1, in addition to findings of edema and a defective or abnormal subchondral geographical area. Additionally, ARCO staging categorized the MRI features of AVN based on the percentage area involvement of femoral head circumference (Table 3).

**Table 3:** ARCO staging approach categories of MRI features based on the percentage area involvement of femoral head circumference. ARCO, Association Research Circulation Osseous; MRI, magnetic resonance imaging

Stages	Percentage
Minimal (A)	<15%
Moderate (B)	15%-30%
Extensive (C)	>30%

## Results

**Figure 1:**
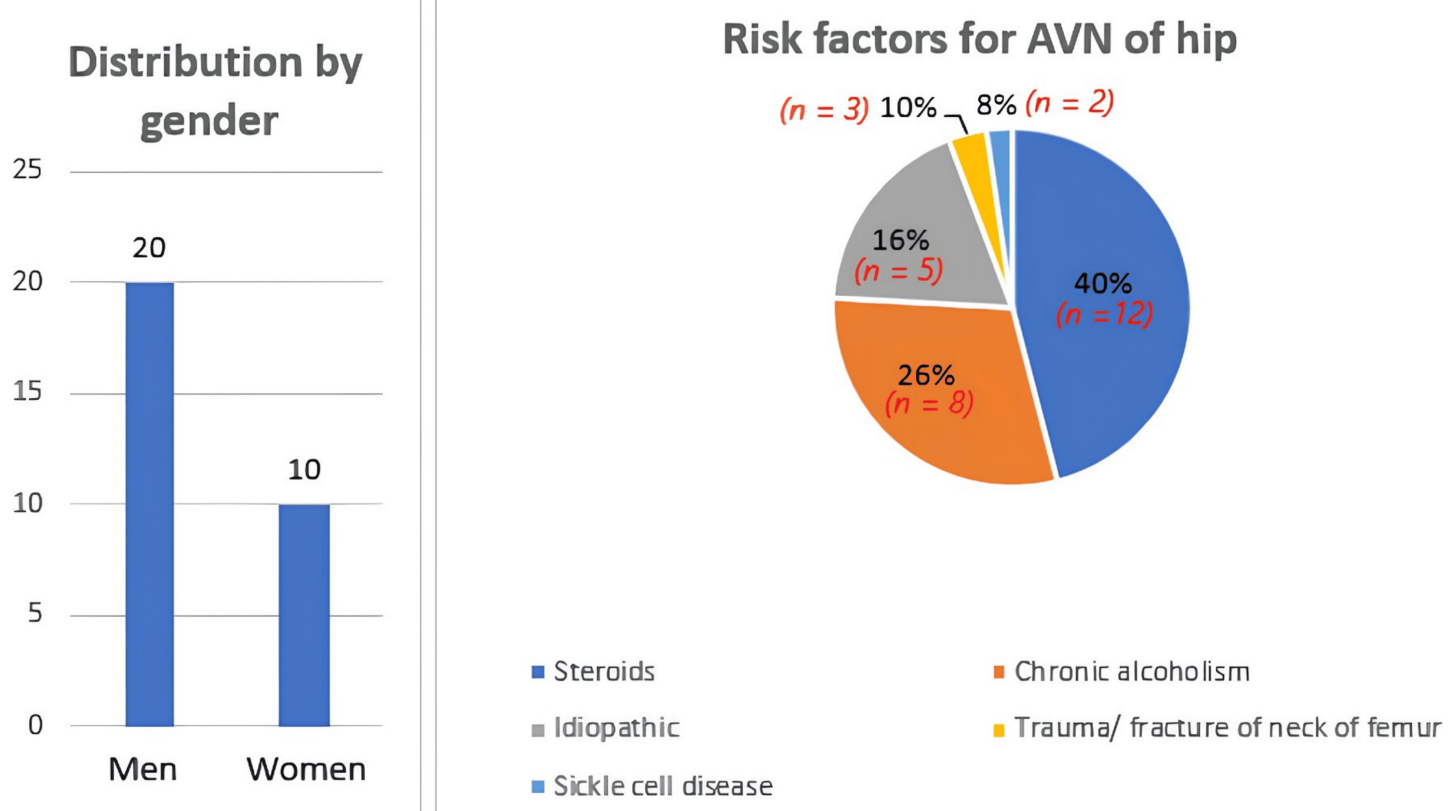
Bar graph and pie chart showing distribution by gender and percentage risk factors for AVN of the hip. AVN: avascular necrosis

We studied 30 patients aged 20-65 years old with a mean age of 45 years. Of these, 66.7% were males (n = 20), and 33.3% were females (n = 10) suspected of femoral head AVN (Figure 1). We studied their hip radiographs and MRI findings.

Forty percent of the patients (n = 12) were on steroids for various reasons, 26.7% (n = 8) were chronic alcoholics, and 16.7% (n = 5) were idiopathic. The other less common causes in our study were a history of trauma or fracture of the neck of the femur (n = 3) and sickle cell disease (n = 2; Figure 1). Of the 45 hips of the 30 patients studied, 15 patients had bilateral disease affecting a total of 30 hips (66.7%), and 15 patients had unilateral disease affecting a total of 15 hips (33.4%). Of the 30 hips (bilateral disease), five (13.3%) contralateral hips were clinically occult and were incidentally diagnosed with stage II AVN. No unilateral disease progressed to bilateral disease over the six-month period.

On radiographic analysis, out of the 45 hips, plain radiography could detect AVN in 28 hips (62.2%), but plain radiography could not detect it in 17 hips (37.8%), which MRI otherwise detected, thereby statistically significant with a p value of <0.001 (Table 4).

**Table 4:** Diagnosis of AVN of the hip by modality. Values represented are percentages (frequency). The test used a chi-square test. *P values of <0.05 are statistically significant. AVN, avascular necrosis; MRI, magnetic resonance imaging

Stages of AVN of the hip (Ficat and Arlet classification)	Percentage (%) and number of hips (n = 45)		P value
	X-ray	MRI	
Stage I	0.0% (n = 0)	4.4% (n = 2)	0.474
Stage II	0.0% (n = 0)	28.9% (n = 13)	<0.001*
Stage III	46.7% (n = 21)	51.1% (n = 23)	0.833
Stage IV	15.6% (n = 7)	15.6% (n = 7)	1

Using MRI, we could diagnose AVN in all 45 hips under evaluation (100%). Of these, 12 hips revealed stage III (early) AVN, and 11 hips revealed stage III (late) AVN according to the ARCO staging system. The MRI diagnosis and the staging of AVN of the hip joint by the ARCO and Ficat and Arlet staging systems were in correlation with each other (Table 5).

**Table 5:** Correlation of MRI features (ARCO staging system) and combined plain X-ray + MRI features (Ficat and Arlet classification). AVN, avascular necrosis; ARCO, Association Research Circulation Osseous; MRI, magnetic resonance imaging

Stages of AVN of the hip	Percentage (%) and number of hips (n)	
	ARCO staging system	Ficat and Arlet classification
Stage I	4.4% (n = 2)	4.4% (n = 2)
Stage II	28.9% (n = 13)	28.9% (n = 13)
Stage III, early (flattening of the femoral head)	Early IIIA, 4.4% (n = 2)	51.1% (n = 23)
	Early IIIB, 8.9% (n = 4)	
	Early IIIC, 13.3% (n = 6)	
Stage III, late (flattening with eventual femoral head collapse)	Late IIIA, 4.4% (n = 2)	
	Late IIIB, 13.3% (n = 6)	
	Late IIIC, 6.7% (n = 3)	
Stage IV	15.6% (n = 7)	15.6% (n = 7)

Cases

Case 1

**Figure 2:**
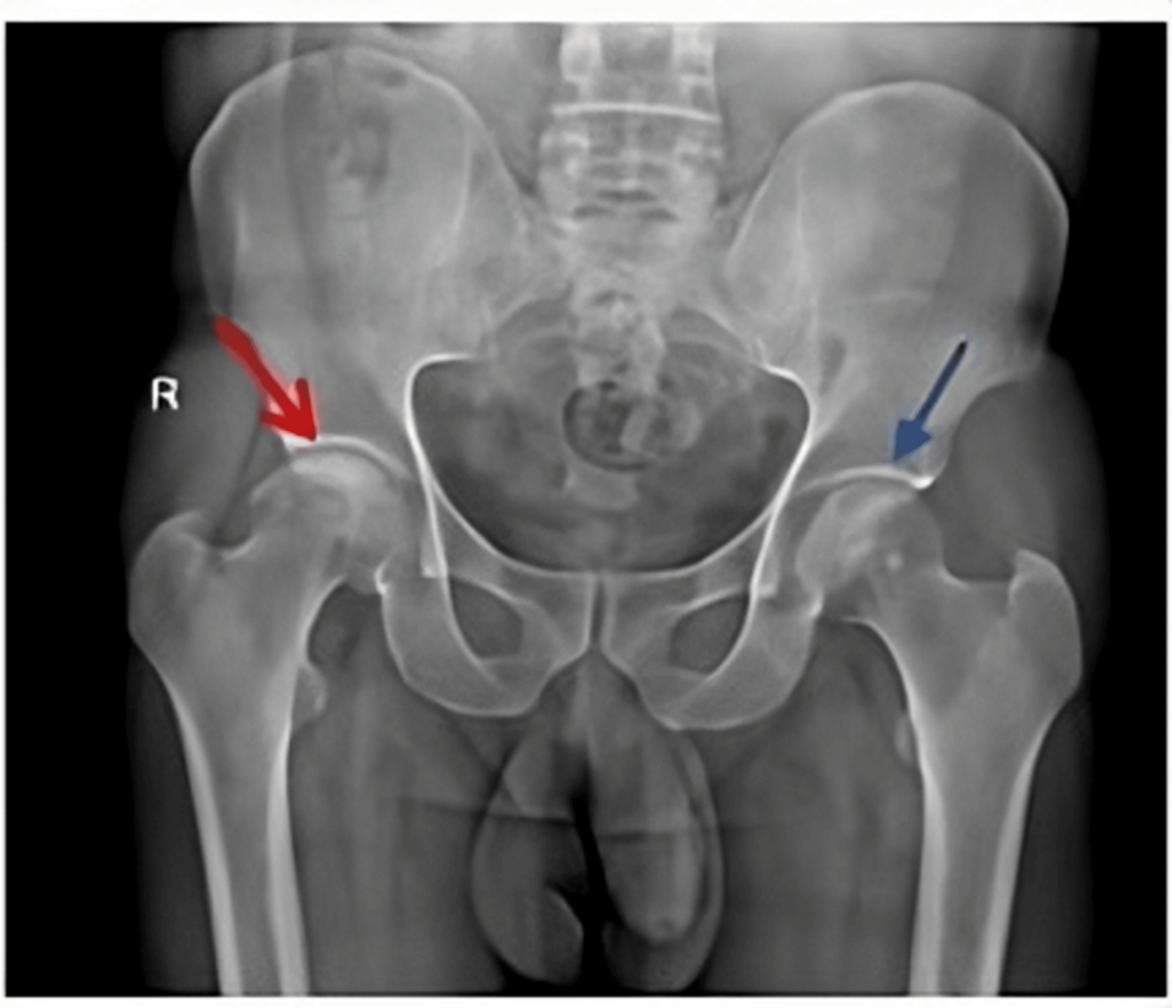
X-ray of the pelvis and both hips of case 1, anteroposterior (AP) view. Findings:Flattening with mild cortical collapse and sclerosis of the right femoral head representing Ficat stage III AVN (red arrow) and minor subarticular sclerosis of the left femoral head representing Ficat stage II AVN (blue arrow). AVN: avascular necrosis

**Figure 3:**
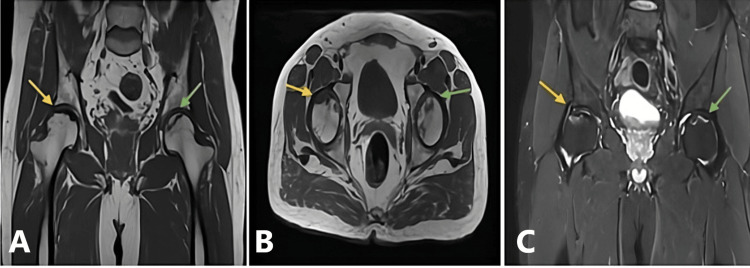
MRI of the pelvis and both hips. Findings:T1W axial and coronal images reveal stage III with the subchondral collapse of the femoral head on the right side (yellow arrow) and Ficat and Arlet stage II with maintained spherical contour of the femoral head on the left side (green arrow) and similarly ARCO stage IIIC (late) on the right side and stage II on the left side. Findings point to AVN of the bilateral femoral heads (A and B). Short tau inversion recovery (STIR) coronal images reveal mild hyperintense marrow edema noted involving the right femoral head (yellow arrow) with no significant marrow edema involving the left femoral head (C; green arrow). T1W, T1-weighted; ARCO, Association Research Circulation Osseous; AVN, avascular necrosis; MRI, magnetic resonance imaging

A 48-year-old male patient arrived at Dr. D. Y. Patil tertiary care hospital, Pimpri, Pune, India, complaining of having had pain in the bilateral hips for three years (right > left). He had a history of steroid intake for COVID-19. The patient was subjected to plain radiography (Figure 2) and an MRI of the pelvis (Figure 3) of both hips.

Case 2

**Figure 4:**
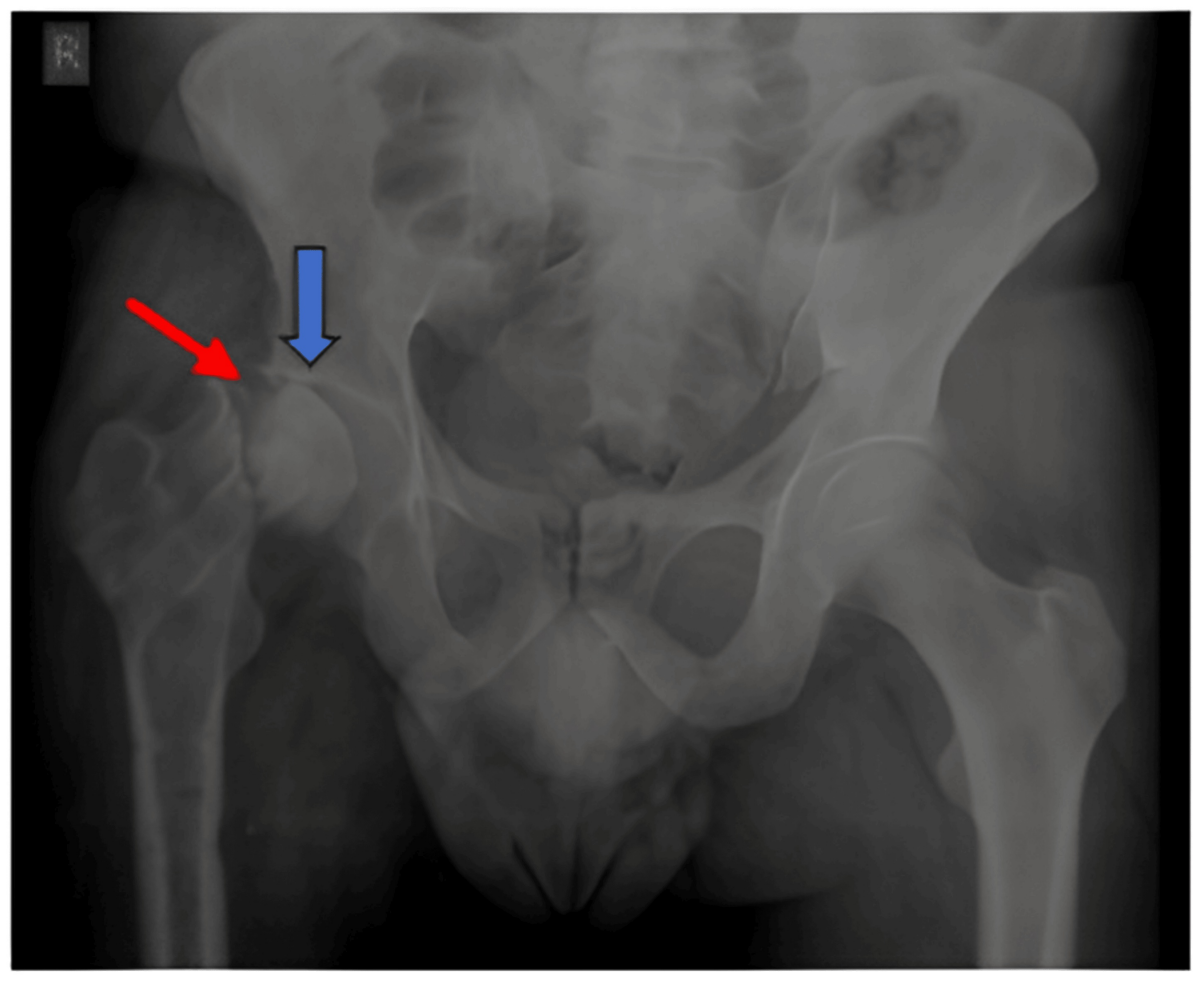
X-ray of the pelvis and both hips of case 2, AP view. Findings:* *A fracture of the neck of the right femur is noted (red arrow) to rule out the nonunion of the fractured neck. Opposed fractured margins show irregular areas of sclerosis. Marginal osteophytes are noted at the fractured right head and acetabulum. The right femoral head shows increased density or sclerosis with a loss of normal contour and secondary degenerative changes, representing Ficat and Arlet stage IV AVN (blue arrow). Increased joint space is noted at the right hip joint. Internal fixation markings are noted at the intertrochanteric region and proximal femur on the right side. AP, anteroposterior; AVN, avascular necrosis

**Figure 5:**
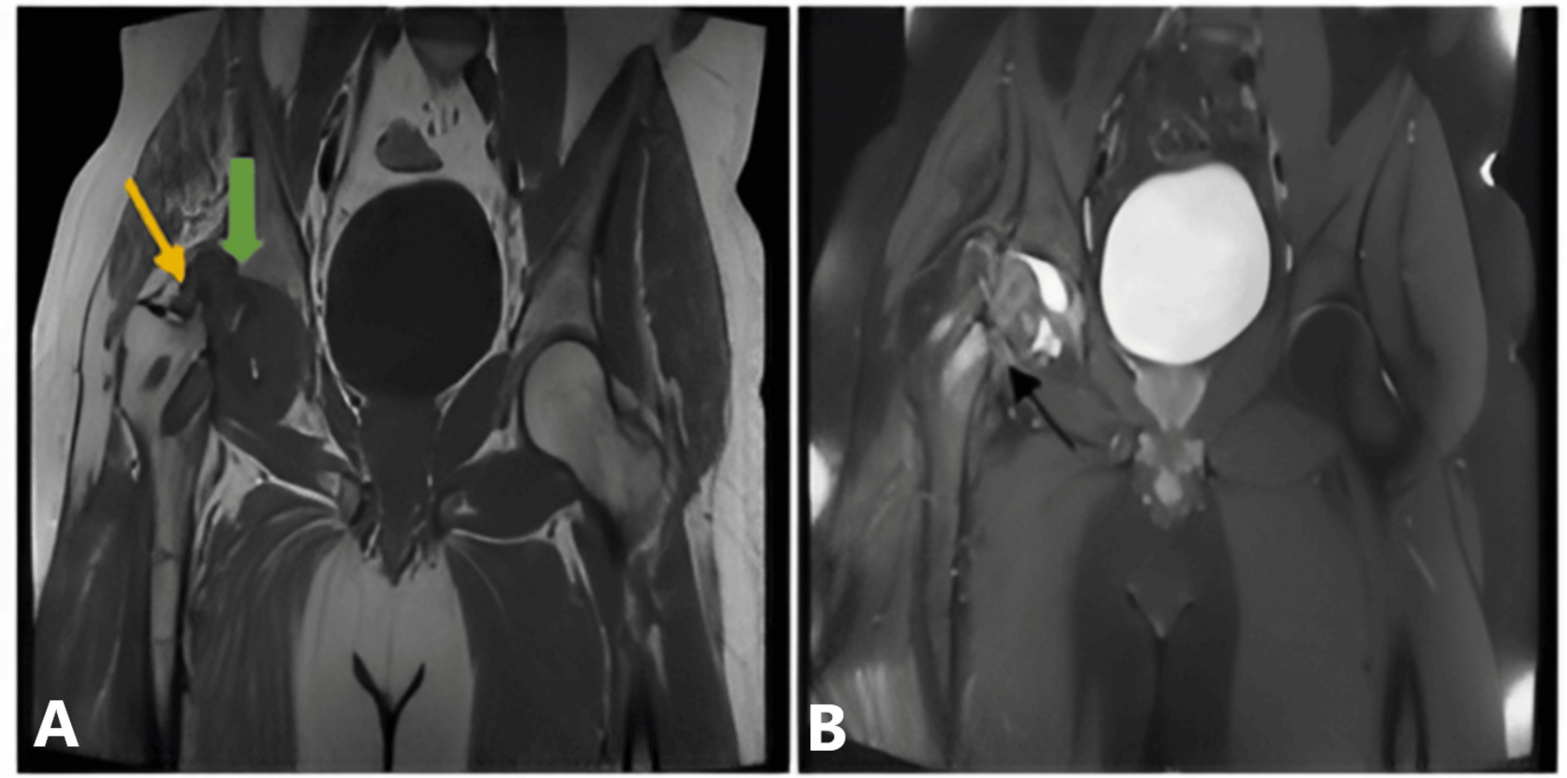
MRI of the pelvis and both hips. Findings:T1W axial and coronal images reveal a fracture of the neck of the right femur (yellow arrow), with signs of previous operative intervention; subluxation at the right hip joint; synovial effusion in the right hip joint; right femoral head AVN involving 70%-80% femoral head circumference (green arrow), with secondary degenerative changes; and Ficat and Arlet stage IV (green arrow) and ARCO stage IV (A). Coronal STIR images reveal significant hyperintense bone marrow edema in the right femoral head, neck, and proximal shaft (B; black arrow). T1W, T1-weighted; AVN, avascular necrosis; ARCO, Association Research Circulation Osseous; MRI, magnetic resonance imaging

A 38-year-old male who had been complaining of right hip pain for two years arrived at Dr. D. Y. Patil tertiary care hospital, Pimpri, Pune, India, with a history of road traffic accident over the previous year resulting in a fractured neck of the right femur. The patient underwent plain radiography (Figure 4) and an MRI of the pelvis of both hips (Figure 5).

Case 3

**Figure 6:**
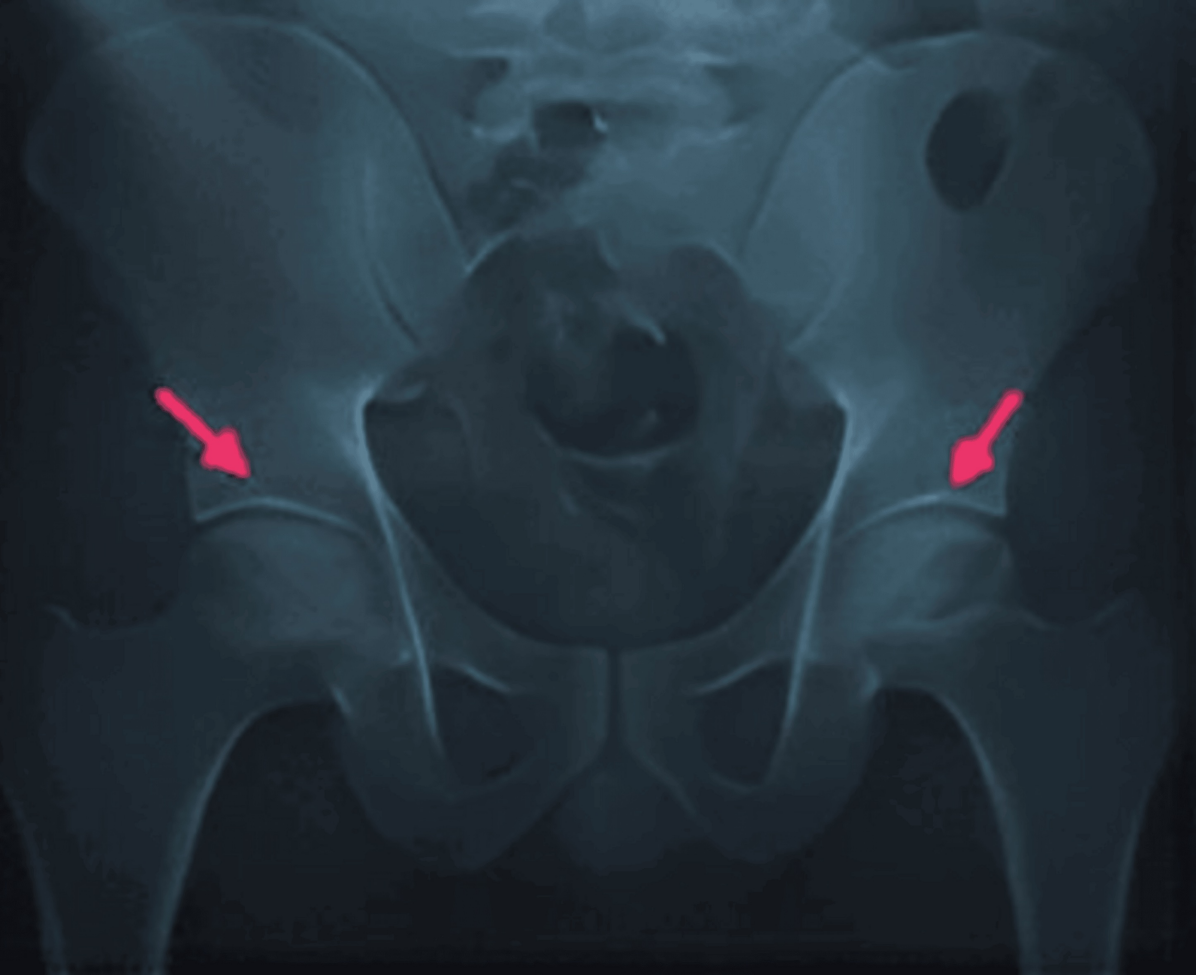
X-ray of the pelvis and both hips of case 3, AP view. Findings: Flattening, cortical collapse, and sclerosis of the bilateral femoral head, representing bilateral Ficat and Arlet stage III AVN (red arrow). AP, anteroposterior; AVN, avascular necrosis

**Figure 7:**
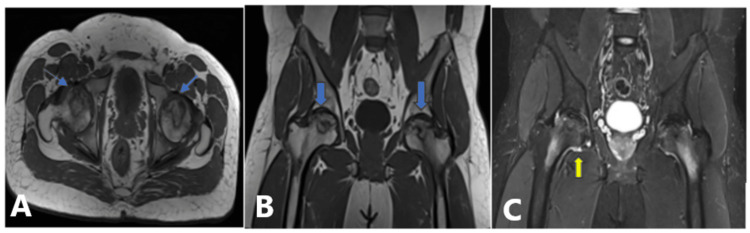
MRI of the pelvis and both hips. Findings:T1W axial and coronal images reveal features of AVN involving bilateral femoral heads in the form of an abnormal subchondral geographical area involving approximately 30%-40% of the bilateral femoral head circumference with the flattening of bilateral femoral heads: Ficat and Arlet stage III and ARCO early stage IIIC on both sides (A and B; blue arrow). STIR coronal images reveal mild hyperintense marrow edema involving bilateral femoral heads with mild right hip joint effusion (C; yellow arrow). T1W, T1-weighted; AVN, avascular necrosis; ARCO, Association Research Circulation Osseous; STIR, short tau inversion recovery; MRI, magnetic resonance imaging

A 32-year-old male patient arrived at Dr. D. Y. Patil tertiary care hospital, Pimpri, Pune, India, complaining of pain in the bilateral hips and difficulty walking for two years. He had a history of chronic alcoholism for 10 years. The patient was subjected to a plain radiograph (Figure 6) and an MRI of the pelvis of both hips (Figure 7).

## Discussion

The prognosis of hip joint AVN depends on its stage at the time of diagnosis. As of now, there has been no agreement on how to treat AVN sufferers. Pharmacotherapy and physical treatment are nonsurgical approaches used for patients in the early precollapse phases of the condition. For patients with advanced illness, surgery is advised [11]. Surgical management depends on various stages of AVN. Management at the early stages includes core decompression and total hip replacement at the late stages. In the early stages, plain radiographs may appear normal, but MRI diagnoses AVN even in the initial stages in the form of marrow edema. Thus, MRI is important for diagnosis and management.

The mean age of cases in this study was 45 years, with 20-65 years as the age range for hip AVN.

The male-to-female ratio was 2:1. In this study, the main symptom was pain, followed by associated limping. In Huang et al.’s study, 89% (n = 98) of 110 hips were painful [12]. Coleman et al. assessed 24 cases of AVN; the risk variables included steroid medication (16 cases), trauma without fracture (four cases), alcohol misuse (one case), and idiopathic disease (three cases) [13]. The most frequent risk factor in our analysis was the use of steroids. In our study, we discovered that patients who had had steroid therapy as a risk factor for developing AVN of the femoral head had it for anywhere between three weeks and two years.

Huang et al. found bone marrow edema in 53 of 110 hips (48.2%) with AVN [12]. In our study, 23 of 45 hips (51.1%) had bone marrow edema. Using MRI, Mitchell et al. detected femoral head flattening in 19 cases; 16 (84.2%) of these were confirmed radiographically [14]. In our study, 15 femoral heads showed flattening; 13 (86.7%) of these were confirmed on radiographs, and the two cases that were not detected on radiographs were diagnosed on MRI. In Coleman et al.’s study, when an MRI of the hip was done in a group of selected patients with persistent pain in their hips, the MRI diagnosed 13 hips with AVN, where plain radiographs were absolutely normal [13]. Our study also showed similar results. Thus, MRI is very sensitive and detects early radiograph-negative cases of AVN of the femoral head by detecting marrow edema with or without associated abnormal subchondral geographical area, which otherwise cannot be detected on plain radiographs. In Khanna et al.’s study, the first set of coronal T1-weighted (T1W) images missed AVN in one hip [15]. In our study, coronal T1W images detected AVN in every case. The majority of the affected hips were Ficat and Arlet stage III (ARCO stage III) AVN (51.1% affected hips), followed by Ficat and Arlet stage II (ARCO stage II) AVN (28.9% affected hips).

Our study has two limitations: a small sample size and limited data. Further studies involving a larger sample size and more data would be helpful in drawing broader conclusions.

## Conclusions

The accuracy of any classification system is significantly important to the proper diagnosis and treatment of AVN of the femoral head.

The aim of our study was to measure the correlation of plain radiography with MRI in assessing AVN of the femoral head. The assessment of AVN features based solely upon plain radiography may fail to reveal any important features of AVN in Ficat and Arlet stages II and III. Due to its multiplanar capability, superior spatial resolution, and better tissue characterization, MRI is very sensitive and enables early and prompt detection of radiograph-negative and clinically unsuspected cases of femoral head AVN.
